# CHALLENGES AND IMPERFECT SOLUTIONS FOR COGNITIVE SCREENING WITH OLDER IMMIGRANTS

**DOI:** 10.1093/geroni/igz038.2590

**Published:** 2019-11-08

**Authors:** Lisa Willoughby, Chuleeporn Pusopa, Sattha Prakobchai, Paweena Meekanon, Spondita Goswami, Hisako Matsuo, Theodore K Malmstrom

**Affiliations:** Saint Louis University, St. Louis, Missouri, United States

## Abstract

Cultural diversity among older adults is increasing and with this comes challenges in health care needs, including the detection of cognitive impairment. Cognitive impairments manifests in many ways, with early symptoms often difficult to detect. Detecting cognitive dysfunction is typically facilitated with brief, portable screening tools. Scores on screening tools may be influenced by culture, education, and verbal abilities; in particular, these are acute issues for screening older immigrants from linguistically and culturally diverse backgrounds. The consequences of improper screening are high and, as such, finding practical, cost-effective solutions is of critical importance. In this project, we qualitatively examined the usability of different cognitive screening tools with the ultimate goal of improving the detection and classification of cognitive dysfunction among older adult immigrants. We extended our previous work by piloting adaptation guidelines for the Saint Louis University Mental Status (SLUMS) exam for use with linguistically and culturally diverse persons. We recruited 23 older immigrants (6 non-English speaking) and 14 U.S. born participants to explore the usability and test-retest reliability of the SLUMS exam administered with and without the adaptation guidelines. Our attempts to improve the flexibility of the SLUMS exam did not achieve the level of success as anticipated. Although this pilot work had low power, when coupled with our past work on developing adaptation guidelines, sheds critical light on the layered complexity that arises at the intersections of education, culture, race, gender, socioeconomic status, and intercultural interactions and the resulting potential directions for future work will be discussed.

